# Development of a Confined-Space Suitability Index (CSSI) using Least Absolute Shrinkage and Selection Operator (LASSO) regression: a pilot study for structured fitness-for-duty screening

**DOI:** 10.1371/journal.pone.0350740

**Published:** 2026-06-02

**Authors:** InHo Lee, SangHee Hong, EunChul Jang, JuneHee Lee, JeongBeom Lee

**Affiliations:** 1 Department of Occupational and Environmental Medicine, Soonchunhyang University Cheonan Hospital, Cheonan, Republic of Korea; 2 Department of Physiology, College of Medicine, Soonchunhyang University, Cheonan, Republic of Korea; 3 Department of Medical Sciences, Graduate School, Soonchunhyang University, Asan, Republic of Korea; 4 Department of Occupational and Environmental Medicine, Soonchunhyang University Hospital, Seoul, Republic of Korea; 5 Advanced Pharmacology and Physiology Integrated Sciences (AXIS), College of Medicine, Soonchunhyang University, Cheonan, Republic of Korea; Korea National University of Transportation, KOREA, REPUBLIC OF

## Abstract

**Background:**

Confined space work exposes workers to complex risks, including oxygen deficiency, hazardous gases, and physical strain, requiring structured criteria for fitness-for-duty screening. This study aimed to develop a Confined-Space Suitability Index (CSSI) based on routinely available health examination and functional test data.

**Methods:**

A total of 111 workers were analyzed. Risk factors were classified into anthropometric and metabolic factors, functional and physiological factors, and lifestyle factors. Low cardiorespiratory fitness was defined using age- and sex-specific VO₂max reference thresholds from Korea Occupational Safety and Health Agency (KOSHA) H-43–2021 and Canadian Public Health Association reference values. Risk-factor weights were derived using LASSO regression, and internal validation was performed using leave-one-out cross-validation and bootstrap stability analysis.

**Results:**

According to the revised CSSI criteria, 86 workers were classified as suitable, 19 as caution, and 6 as unsuitable. The independent specialist assessment classified 7 workers as unsuitable. In leave-one-out cross-validation, the CSSI showed an AUROC of 0.940 and an AUPRC of 0.567, with sensitivity of 0.833, specificity of 0.933, and negative predictive value of 0.990. In bootstrap stability analysis, dyslipidemia, low cardiorespiratory fitness, and hypertension were consistently selected.

**Conclusions:**

The CSSI may serve as a structured screening and referral-support index for identifying workers who require additional specialist evaluation before confined space work. However, it should be interpreted as an auxiliary index rather than a replacement for specialist fitness-for-duty judgment.

## Introduction

Working in a confined space poses a significant health risk to workers due to limited ventilation and the constant threat of hazardous gas buildup or oxygen deficiency [1,2]. From 2011 to 2017 in the U.S. alone, 882 fatalities and injuries were reported from confined space accidents [2]. The fatality rate for these incidents is significantly higher than for general workplace accidents [1].

Asphyxiation and poisoning are leading causes of death in these environments [1], and a sudden health issue can have fatal consequences. For example, acute cardiac events account for 45% of all on-duty deaths for special workers like firefighters [3,4]. If such a cardiovascular emergency occurs in a confined space, delayed rescue can make the situation even more lethal. Therefore, workers entering confined spaces need to be evaluated not only for environmental risks such as oxygen deficiency and harmful gases, but also for personal health conditions such as cardiopulmonary function, metabolic status, and sensory function.

Most countries now have institutionalized fitness-for-work evaluations to determine if a worker can perform their duties without endangering their own or others’ health and safety [5]. However, these guidelines often focus mainly on general check-ups and do not fully account for the specific risks of certain jobs, especially those in confined spaces [6]. Sensory and physical factors, such as hearing, have a strong connection to industrial accidents [7,8]. However, current work suitability assessments often rely primarily on questionnaires and basic tests, indicating a limitation in structured evaluation systems that consistently reflect the physiological burden of high-risk tasks [9].

In confined spaces, adaptability of the respiratory and circulatory systems is critical due to limited air supply and the use of respiratory protective equipment. There is accumulating evidence that standard pulmonary function tests and interviews alone are insufficient to screen for all high-risk situations [10]. In high-risk jobs, the need for function-based evaluation such as exercise load testing has been raised because it is difficult to fully predict the cardiopulmonary load that may occur during work only with resting tests [11]. There are also reports that autonomic nervous system indicators such as heart rate variability may help to assess job stress and physiological adaptation status [12]. However, there are practical limitations in terms of standardization of testing methods, access to equipment, and cost when applying these precise indicators to all actual workplaces [13]. Therefore, although the existing work suitability assessment system considers both health risks and work performance capacity [5], there are still not enough structured screening tools that can practically reflect the cardiopulmonary function, sensory function, metabolic stability, and emergency response capacity required when entering a confined space [6,9].

Confined-space work has complex risk conditions different from general work. Limited ventilation, high-temperature environments, physical burden due to wearing protective gear, and the possibility of delayed escape in emergency situations may exist at the same time. The Work Ability Index (WAI) has been widely used to evaluate workers’ overall work ability, but it has a limitation in that much of it is based on self-reported responses [14]. In particular, in confined space work, hearing for alarm recognition and communication, cardiopulmonary function to tolerate wearing protective gear, blood pressure stability under load conditions, and metabolic disease-related risks must be considered together, so it is difficult to fully reflect these job-specific needs using only general work ability tools. Recently, machine learning has been used to predict occupational risks. This makes it possible to design scoring systems based on objective health data [15]. The WAI still provides a useful conceptual background [16], but confined space tasks require more direct indicators of physiological and sensory function. The need to adapt tests to specific job demands has been pointed out for many years. One example is functional capacity testing in physical therapy, which is often used in screening before job placement [17].

There has also been an ongoing discussion about the necessity of updating cardiopulmonary assessments for workers who use respiratory protection [18]. Various other studies further support the validity and feasibility of using job-specific metrics, including evaluations of the effectiveness of medical judgments [19], the historical basis for medical evaluations before using respiratory protection [20], guidelines for screening low back pain risks in jobs involving heavy lifting [21], analysis of reasons for unsuitability among maritime workers [22], and the development of safety tools for confined spaces [23]. However, comprehensive work suitability evaluation conducted by specialists is time-consuming and expensive, and is difficult to access easily from all workplaces. Therefore, an objective screening tool is needed that can systematically identify workers who need additional specialist evaluation using general health examination and functional test data.

Against this backdrop, this study attempted to develop a Confined-Space Suitability Index (CSSI) for screening confined space work suitability by integrating objective health examination and functional test variables such as metabolic indicators, blood pressure, pulmonary function, hearing, electrocardiographic findings, and cardiorespiratory fitness. This approach reflects physiological criteria consistent with rigorous medical standards for high-risk occupational groups [24]. In this preliminary study, CSSI was not designed as a tool to replace a specialist’s final work suitability judgment, but as a screening and referral-support index to summarize the major physiological and clinical risk areas considered in the specialist evaluation process with structured weighted scores [11,13].

### Goal and significance of the pilot study

This study is an exploratory preliminary study to develop LASSO-based CSSI that supports screening for confined space work suitability using data from 111 workers. CSSI included general health examination and functional test variables such as pure-tone audiometry, pulmonary function testing, electrocardiographic assessment, blood pressure, metabolic indicators, and VO₂max-based cardiorespiratory fitness. The purpose of this study is not to validate CSSI as a definitive clinical judgment tool, but to explore whether the weighted scoring approach can provide a structured framework to identify high-risk workers who need additional specialist fitness-for-duty evaluation.

Despite the limited number of samples, this study explored the applicability of weighted score-based indicators that select high-risk workers before working in confined spaces using standardized health examination and functional test data. CSSI has the potential to be used as a practical screening framework to assist pre-placement health management and referral decision-making in workplaces where immediate access to specialist evaluation is difficult. However, in order to apply CSSI to a wider industrial health field, external verification studies including larger scale and various working environments are needed.

## Method

### Study design and participants

This study was designed to assess workers’ suitability for high-risk confined space work. The evaluation protocol was developed to reflect the major environmental stressors identified in the ‘worst-case’ work scenario, such as high temperature and humidity, poor ventilation, excessive physiological load from wearing respiratory protective equipment, including self-contained breathing apparatus (SCBA), and potential exposure to harmful substances.

The participants were 111 workers selected from an initial pool of 143. We excluded four individuals who were deemed unsuitable during preliminary screening (e.g., psychiatric history) and 28 who did not complete the tests or consent to the evaluation. Consequently, the final analysis included 111 adult workers who proceeded to the comprehensive specialist evaluation.

### Determination of fitness-for-duty (Reference standard)

In this study, the specialist fitness-for-duty judgment was used as an independent clinical reference for comparison with the CSSI results. This judgment was independently performed by occupational and environmental medicine (OEM) specialists before calculating the CSSI score, and it was clarified that CSSI was developed as an auxiliary indicator for selecting workers who needed specialist evaluation, not a model that replaces the specialist’s judgment. The specialists conducted a comprehensive fitness-for-duty evaluation based on rigorous industrial hygiene standards for confined spaces and fire brigade members (referencing NFPA 1582) [24]. The evaluation protocol included: 1) Assessment of medical history regarding tolerance to respiratory protective equipment. 2) Review of physiological resilience under simulated “worst-case scenarios.” 3) Functional capacity testing, including a 3-minute step test simulating the weight of protective gear to assess cardiovascular recovery and physical endurance.

Workers who were medically unable to safely perform essential tasks or showed meaningful safety risks under load conditions were classified as unsuitable in the specialist evaluation (n = 7). This independent specialist judgment was used as a clinical reference for comparison with the CSSI-based classification results, and the CSSI was not interpreted as a final judgment tool to replace the specialist judgment.

### Classification of variables and risk factors

For this study, risk factors for confined space work suitability were categorized into three tiers based on occupational health and clinical guidelines. All variables were coded as dichotomous (0 = not applicable, 1 = applicable). The risk factors were summed within each tier and across all tiers to produce tier-specific and overall risk totals. These variables were selected to reflect cardiopulmonary function, sensory function, metabolic stability, and lifestyle-related risk factors relevant to confined space work, and then structured into three stages of risk factors for calculating CSSI.

Binary risk factor coding was used for the purpose of simplifying the evaluation procedure when applied in the field and enabling repeatable screening according to the same criteria. However, this coding was not intended to replace the final clinical judgment, but was used as a structured approach to select workers who needed further evaluation [25].

### Tier 1: Anthropometric and metabolic risk factors

This tier includes basic health metrics that influence basal metabolic rate, heat tolerance, and physical mobility, which are critical in confined spaces characterized by poor ventilation and restricted movement. Each variable was defined based on established industrial and clinical guidelines.

1) Obesity: Defined as a body mass index (BMI) of ≥25 kg/m² or waist circumference of ≥90 cm for men and ≥85 cm for women, following Korean guidelines. These cutoff points are validated for their association with metabolic disorders and reduced physical mobility in narrow spaces [26].
2) Hypertension: A risk was assigned for a systolic blood pressure of ≥140 mmHg, a diastolic blood pressure of ≥90 mmHg, or the use of antihypertensive medication. Uncontrolled hypertension poses a risk of sudden incapacitation under physical stress [27].
3) Diabetes: Workers were classified as a risk if their fasting blood glucose was ≥ 126 mg/dL, their HbA1c was ≥ 6.5%, or if they were taking antidiabetic medication. Poor glycemic control can lead to dizziness or consciousness loss during strenuous work [28].
4) Dyslipidemia: Risk was classified for low-density lipoprotein cholesterol (LDL-C) ≥160 mg/dL, high-density lipoprotein cholesterol (HDL-C) <40 mg/dL, total cholesterol ≥240 mg/dL, or the use of lipid-lowering medication [27,29].
5) Elevated Liver Enzymes: A risk was coded if any of the following values exceeded the normal upper limit: AST (Aspartate aminotransferase) > 40 IU/L, ALT (Alanine aminotransferase) > 40 IU/L, or γ-GTP (Gamma-glutamyl transferase) > 50 IU/L [30,31].
6) Resting heart rate (HR): A normal resting HR was considered to be within the 50–100 bpm range. Bradycardia (<50 bpm) and tachycardia (>100 bpm) were both deemed risk factors indicating potential cardiovascular instability [24,25].
7) Anemia: Anemia was defined as a hemoglobin (Hb) level <13 g/dL for men and <12 g/dL for women. In confined spaces with potential oxygen deficiency, anemia critically reduces oxygen transport capacity, increasing the risk of hypoxia [32].

### Tier 2: Functional and physiological risk factors

This tier assesses the functional capacity required to operate safety equipment (e.g., respirators) and respond to emergencies.

1) Hearing Loss: A hearing loss was classified as a risk if the average pure-tone audiometry value (0.5–4 kHz) was > 25 dB. This threshold considers the critical need to hear gas alarms and communicate via radio in noisy confined environments [33].
2) Abnormal Pulmonary Function: This was classified as a risk if FEV₁/FVC was < 0.70 or FEV₁ was < 80% of the predicted value. Adequate lung function is essential for wearing self-contained breathing apparatus (SCBA) without respiratory distress [34,35].
3) Abnormal ECG: Any abnormal findings on a resting or post-exercise ECG (excluding normal sinus bradycardia) were considered a risk. Given the high cardiac demand of confined space rescue scenarios, strict cardiovascular screening was applied, referencing standards like NFPA 1582 [25].
4) Maximal oxygen uptake (VO₂max): Low cardiorespiratory fitness was defined as less than the lower limit of the age- and sex-specific normal range of VO₂max presented in the Korea Occupational Safety and Health Agency (KOSHA) H-43–2021 guideline and Canadian Public Health Association reference values [36,37].

### Tier 3: Lifestyle factors

This tier addresses lifestyle habits that can exacerbate physiological strain under extreme conditions.

1) Smoking History: A risk was defined as a cumulative history of smoking of ≥20 pack-years, regardless of current status. Long-term smoking compromises lung function and carbon monoxide diffusion capacity, reducing tolerance to potential hypoxia in confined spaces [38].
2) Habitual Alcohol Consumption: Habitual alcohol consumption was classified as a risk when alcohol drinking occurred two or more times per week. Chronic alcohol use can impair thermoregulation and increase susceptibility to dehydration and heat stroke in hot, enclosed environments [39].

### Weighting and Least Absolute Shrinkage and Selection Operator (LASSO) regression

To complement the simple count of risk factors, LASSO regression analysis was used to estimate the relative contribution of risk factors associated with the CSSI-defined unsuitable classification. The dependent variable of the LASSO model was the dichotomous unsuitable classification defined by the CSSI rule-based category (unsuitable = 1, suitable/caution = 0). The CSSI-based classification categorized workers as suitable, caution, and unsuitable according to the total number of risk factors, and the CSSI-based rule-based category defined 0–3 total risk factors as suitable, 4–5 as caution, and 6 or more as unsuitable. In the independent specialist’s judgment, 7 workers were classified as unsuitable. This result was used as a criterion for comparison with the CSSI-based classification, and was used as a basis for showing that CSSI should be interpreted as an auxiliary indicator for screening and referral, not as a tool that completely replaces the specialist’s judgment. Age and sex were included as covariates in the model. Missing values were replaced with mean values, and all variables were standardized using the z-score method. Among the coefficients calculated by LASSO, only positive coefficients were used in the calculation of the additive CSSI weight. Since a negative coefficient can make certain risk factors interpreted as protective factors, it was treated as 0 for consistency in the interpretation of the screening index. The binary risk factor values of each worker were multiplied by a normalized weight and then summed to calculate the individual raw score. After that, the raw score was converted into the range of 0–100 to calculate the additive CSSI score. The higher the CSSI score, the more the worker was interpreted as having a risk factor combination close to the unsuitable or high-risk category in the CSSI criteria. This score was not used to replace the final judgment of a specialist, but was used as an auxiliary index to select workers who needed additional work suitability evaluation.

### Analysis procedure

Statistical analysis was performed using Python (version 3.13). Pandas and numpy libraries were used for data preprocessing. Descriptive statistics were calculated using scipy and statsmodels. The scikit-learn library was used for z-score standardization, mean imputation, and building the regression model. After calculating the LASSO-based weight, the internal discrimination performance was evaluated by using leave-one-out cross-validation. In addition, bootstrap stability analysis was performed 1,000 times to check how often each risk factor was selected from repeated resampling data. This analysis was performed to further confirm the stability of the model in this preliminary study, where the number of samples and unsuitable cases was limited. Since the CSSI-defined unsuitable classification was a dichotomous outcome, L1-penalized logistic regression was used. Missing values were replaced with mean values, and continuous variables and covariates were standardized by the z-score method. Model performance was evaluated by using the area under the receiver operating characteristic curve (AUROC), the area under the precision–recall curve (AUPRC), sensitivity, specificity, positive predictive value, negative predictive value, and balanced accuracy.

### Ethical considerations

This study analyzed retrospective records collected from May 2021 to April 2024. Data access for analysis was made on June 15, 2024. Since this study was a retrospective study using de-identified data, there was no direct contact with the study subjects. Minors were not included. The study protocol was approved by the Institutional Review Board of Soonchunhyang University Cheonan Hospital (approval number: SCHCA IRB 2024-05-042). The IRB waived the requirement for informed consent in consideration of the retrospective nature of the study and the anonymity of the data. All research procedures complied with the ethical principles of the Declaration of Helsinki (1964) and its subsequent revisions.

## Results

### Demographic characteristics

A total of 111 workers were included in the analysis: 72 males (64.9%) and 39 females (35.1%). The mean age was 44.3 ± 10.1 years and was comparable between men and women. In anthropometric and metabolic indicators, men generally had higher mean BMI, waist circumference, systolic and diastolic blood pressure, triglyceride levels, liver enzyme levels, smoking exposure, and drinking frequency than women, whereas women tended to have higher HDL cholesterol (Table 1). These findings indicate sex-related differences in anthropometric characteristics, lifestyle exposures, metabolic profiles, and hematologic indicators.

**Table 1:** Baseline clinical, functional, and lifestyle characteristics of study participants.

Variable	Unit	Total (N = 111)	Male (N = 72)	Female (N = 39)
Age	years	44.3 ± 10.1	44.5 ± 10.7	44.0 ± 8.9
Body mass index	kg/m²	24.3 ± 3.1	25.0 ± 2.9	23.0 ± 3.2
Waist circumference	cm	79.4 ± 10.3	83.9 ± 8.1	71.1 ± 8.9
Systolic blood pressure	mmHg	121.3 ± 11.0	123.8 ± 10.4	116.7 ± 10.8
Diastolic blood pressure	mmHg	74.7 ± 9.3	76.7 ± 9.0	71.0 ± 8.8
Resting heart rate	beats/min	65.5 ± 9.8	64.6 ± 10.1	67.1 ± 9.3
Fasting glucose	mg/dL	96.3 ± 10.9	97.2 ± 8.7	94.5 ± 14.1
HbA1c	%	5.7 ± 0.4	5.7 ± 0.4	5.7 ± 0.5
Total cholesterol	mg/dL	202.9 ± 34.6	198.9 ± 36.7	210.3 ± 29.5
LDL cholesterol	mg/dL	122.4 ± 31.8	119.4 ± 33.0	127.9 ± 28.9
HDL cholesterol	mg/dL	57.9 ± 15.7	54.7 ± 15.6	63.9 ± 14.2
Triglycerides	mg/dL	135.9 ± 91.6	150.0 ± 103.8	109.9 ± 55.5
AST	IU/L	25.5 ± 14.4	27.6 ± 16.0	21.7 ± 10.0
ALT	IU/L	30.0 ± 28.4	35.0 ± 32.8	20.7 ± 13.8
γ-GTP	IU/L	27.0 ± 21.6	33.4 ± 23.2	15.2 ± 10.9
Hemoglobin	g/dL	14.8 ± 1.5	15.6 ± 1.0	13.3 ± 1.0
Pure-tone average, 0.5–4 kHz	dB	13.8 ± 8.0	15.1 ± 9.1	11.5 ± 4.6
FEV1/FVC	%	84.0 ± 6.0	84.4 ± 6.4	83.3 ± 5.0
FEV1 predicted	%	88.1 ± 8.7	88.0 ± 9.2	88.2 ± 7.8
VO₂max	mL/kg/min	32.5 ± 5.5	34.5 ± 5.3	28.8 ± 3.6
Smoking exposure	pack-years	5.0 ± 9.7	7.8 ± 11.2	0.0 ± 0.0
Alcohol drinking frequency	times/week	1.0 ± 1.2	1.3 ± 1.2	0.3 ± 0.7

Continuous variables are presented as mean ± standard deviation. HbA1c, glycated hemoglobin; LDL, low-density lipoprotein; HDL, high-density lipoprotein; AST, aspartate aminotransferase; ALT, alanine aminotransferase; γ-GTP, gamma-glutamyl transpeptidase; FEV1, forced expiratory volume in 1 second; FVC, forced vital capacity; VO₂max, maximal oxygen uptake.

### Distribution of risk factors

The frequency distribution of individual risk factors is presented in Table 2. The most common risk factors of all workers were obesity in 43 workers (38.7%), dyslipidemia in 33 workers (29.7%), and low cardiorespiratory fitness in 28 workers (25.2%), and elevated liver enzymes and habitual alcohol consumption were confirmed in 27 workers (24.3%), respectively. By sex, obesity, hypertension, dyslipidemia, elevated liver enzymes, low cardiorespiratory fitness, significant smoking history, and habitual alcohol consumption were more frequently observed in men, and anemia was relatively more frequent in women. These results indicate that the distribution of metabolic, physiological, and lifestyle-related risk factors differed according to sex.

**Table 2:** Tiered operational definitions and distribution of CSSI risk factors.

Tier	Risk factor variable	Operational cutoff	Total (N = 111)	Male (N = 72)	Female (N = 39)
Tier 1: Anthropometric and metabolic risk factors	Obesity	BMI ≥ 25 kg/m² or waist circumference ≥90 cm for men/ ≥ 85 cm for women	43 (38.7)	33 (45.8)	10 (25.6)
Tier 1: Anthropometric and metabolic risk factors	Hypertension	SBP ≥ 140 mmHg, DBP ≥ 90 mmHg, or antihypertensive medication	19 (17.1)	16 (22.2)	3 (7.7)
Tier 1: Anthropometric and metabolic risk factors	Diabetes	Fasting glucose ≥126 mg/dL, HbA1c ≥ 6.5%, or antidiabetic medication	5 (4.5)	3 (4.2)	2 (5.1)
Tier 1: Anthropometric and metabolic risk factors	Dyslipidemia	LDL-C ≥ 160 mg/dL, HDL-C < 40 mg/dL, total cholesterol ≥240 mg/dL, or lipid-lowering medication	33 (29.7)	26 (36.1)	7 (17.9)
Tier 1: Anthropometric and metabolic risk factors	Elevated liver enzymes	AST > 40 IU/L, ALT > 40 IU/L, or γ-GTP > 50 IU/L	27 (24.3)	22 (30.6)	5 (12.8)
Tier 1: Anthropometric and metabolic risk factors	Abnormal resting heart rate	Resting heart rate <50 or >100 beats/min	3 (2.7)	2 (2.8)	1 (2.6)
Tier 1: Anthropometric and metabolic risk factors	Anemia	Hemoglobin <13 g/dL for men or <12 g/dL for women	3 (2.7)	0 (0.0)	3 (7.7)
Tier 2: Functional and physiological risk factors	Hearing loss	Pure-tone average at 0.5–4 kHz > 25 dB	11 (9.9)	10 (13.9)	1 (2.6)
Tier 2: Functional and physiological risk factors	Abnormal pulmonary function	FEV1/FVC < 70% or FEV1 < 80% predicted	20 (18.0)	13 (18.1)	7 (17.9)
Tier 2: Functional and physiological risk factors	Abnormal electrocardiographic finding	Any abnormal ECG finding except normal sinus bradycardia	15 (13.5)	11 (15.3)	4 (10.3)
Tier 2: Functional and physiological risk factors	Low cardiorespiratory fitness	VO₂max below the age- and sex-specific lower bound of the normal category according to KOSHA H-43–2021 and Canadian Public Health Association reference values	28 (25.2)	27 (37.5)	1 (2.6)
Tier 3: Lifestyle factors	Significant smoking history	Smoking exposure ≥20 pack-years	9 (8.1)	9 (12.5)	0 (0.0)
Tier 3: Lifestyle factors	Habitual alcohol consumption	Alcohol drinking ≥2 times per week	27 (24.3)	25 (34.7)	2 (5.1)

BMI, body mass index; SBP, systolic blood pressure; DBP, diastolic blood pressure; LDL-C, low-density lipoprotein cholesterol; HDL-C, high-density lipoprotein cholesterol; AST, aspartate aminotransferase; ALT, alanine aminotransferase; γ-GTP, gamma-glutamyl transferase; ECG, electrocardiogram; VO₂max, maximal oxygen uptake; CSSI, Confined-Space Suitability Index.

### Relative contribution of risk factors (LASSO Weights)

To determine how much each risk factor contributes to CSSI-based unsuitable classification, L1-penalized logistic regression was used (Table 3). As a result of normalizing the positive coefficients calculated from LASSO and converting them to additive CSSI weights, dyslipidemia (0.296), low cardiorespiratory fitness (0.158), abnormal resting heart rate (0.149), hypertension (0.146), and ECG abnormality (0.114) showed the highest weights. On the other hand, significant smoking history, diabetes, anemia, and hearing loss were calculated with an additive CSSI weight of 0 in the final model. These results show that factors related to metabolic risk, cardiorespiratory fitness, and cardiovascular stability acted as major weighted components in the CSSI-based high-risk classification.

**Table 3:** LASSO-derived positive coefficients and normalized additive weights used for CSSI score calculation.

Risk factor	LASSO logistic coefficient	Normalized additive CSSI weight
Dyslipidemia	6.075	0.296
Low cardiorespiratory fitness	3.242	0.158
Abnormal resting heart rate	3.063	0.149
Hypertension	2.992	0.146
Abnormal electrocardiographic finding	2.343	0.114
Habitual alcohol consumption	1.030	0.050
Abnormal pulmonary function	0.730	0.036
Elevated liver enzymes	0.586	0.029
Obesity	0.465	0.023

Only risk factors with positive LASSO coefficients used for calculating the additive CSSI score are shown. Negative or zero coefficients were assigned a weight of 0 to avoid interpreting a risk factor as protective in the screening index. Significant smoking history, diabetes, anemia, and hearing loss had zero additive CSSI weights in the final model.

### CSSI score distribution by CSSI-based fitness category

**Fig 1:**
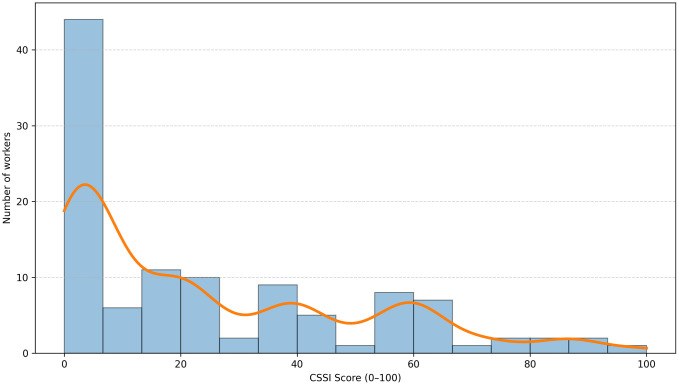
Distribution of LASSO-weighted additive Confined-Space Suitability Index (CSSI) Scores. The histogram shows the distribution of additive CSSI scores normalized to a 0–100 scale. The overlaid curve represents a kernel density estimate for visualization. The additive CSSI score had a right-skewed distribution, with most workers concentrated in the low-to-moderate score range and CSSI-defined unsuitable workers located in the upper tail.

The LASSO-weighted additive CSSI score was normalized in the range of 0–100. Most of the workers were concentrated in the low score range, and the CSSI-based unsuitable group formed a distinct cluster in the upper tail area. In the final CSSI-based classification, workers were classified into suitable, caution, and unsuitable according to the pre-defined total risk factor score criterion. As shown in Fig 1, the weighted CSSI score showed a pattern in which CSSI-based unsuitable workers were separated in the upper tail area.

In the final CSSI rule-based category, which applied the age- and sex-specific VO₂max criteria from KOSHA H-43–2021 and Canadian Public Health Association reference values, 86 workers (77.5%) were classified as suitable, 19 people (17.1%) were classified as caution, and 6 workers (5.4%) were classified as unsuitable. The independent specialist assessment classified 7 workers as unsuitable. The CSSI-defined unsuitable group had multiple metabolic and cardiorespiratory function-related risk factors such as dyslipidemia, low cardiorespiratory fitness, abnormal resting heart rate, hypertension, and abnormal ECG findings. The LASSO weighted additive CSSI score was normalized in the range of 0–100, and the overall mean was 25.3 ± 25.7 points and the median was 17.9. The CSSI score increased stepwise across the suitable, caution, and unsuitable groups: 14.6 ± 15.4, 54.7 ± 13.5, and 86.9 ± 8.6 points, respectively. Fig 1 shows the distribution of additive CSSI scores. Most workers were located in the low-to-moderate score range, whereas CSSI-defined unsuitable workers were concentrated in the upper tail. This distribution indicates that the CSSI score separated high-risk workers into the upper tail of the score distribution. The individual risk-factor profiles of CSSI-defined unsuitable workers are provided in S1 Table in S1 File.

### Internal validation

In leave-one-out cross-validation, the revised CSSI showed discrimination performance for CSSI-defined unsuitable classification, with an AUROC of 0.940 and an AUPRC of 0.567 (Table 4). Sensitivity was 0.833, specificity was 0.933, and balanced accuracy was 0.883. The positive predictive value was limited at 0.417, whereas the negative predictive value was high at 0.990. This suggests that the CSSI should not be interpreted as a final fitness-for-duty decision tool, but rather as a screening-oriented index for excluding workers with a low probability of unsuitability and identifying those who may require additional specialist evaluation.

**Table 4:** Internal validation performance of the revised CSSI using leave-one-out cross-validation.

Metric	Value
N	111
CSSI-defined unsuitable cases	6
AUROC	0.940
AUPRC	0.567
Accuracy	0.928
Balanced accuracy	0.883
Sensitivity	0.833
Specificity	0.933
Positive predictive value	0.417
Negative predictive value	0.990
F1-score	0.556
Threshold	0.50
True positives	5
False positives	7
True negatives	98
False negatives	1

Performance was evaluated using leave-one-out cross-validation. The outcome was CSSI-defined unsuitable classification. AUROC, area under the receiver operating characteristic curve; AUPRC, area under the precision–recall curve.

### Bootstrap stability

In the bootstrap stability analysis, dyslipidemia, low cardiorespiratory fitness, hypertension, habitual alcohol consumption, and ECG abnormalities were consistently selected from repeated resampling data. The frequency of selection was 0.981, 0.976, 0.952, 0.928, and 0.850, respectively. This indicates that the main weighted components of CSSI remained relatively consistent even though the number of unsuitable cases was limited in this preliminary study. Detailed bootstrap stability analysis results are presented in S2 Table in S1 File.

## Discussion

In this study, a weighted CSSI was calculated for 111 workers by integrating anthropometric measures, metabolic indicators, functional tests, and lifestyle factors. Unlike conventional approaches that rely on subjective scoring or simple checklists, this study attempted to develop and internally validate a weighted CSSI that can structurally select workers who need additional specialist evaluation before working in confined spaces based on standard health check-up and functional test data. The CSSI-defined unsuitable group showed a combination of metabolic risk, low cardiorespiratory fitness, cardiovascular stability-related factors, and lifestyle factors (S1 Table in S1 File). In the independent specialist assessment, 7 workers were classified as unsuitable, indicating a slight difference between the CSSI-based classification and the specialist judgment. This supports the concept that confined space work suitability cannot be determined by a single indicator, and a comprehensive evaluation of workers’ physiological reserves is required.

The results of this study show that it is difficult to explain unsuitability or high-risk classification in confined space work with only a specific test. In the high-risk group, metabolic disease-related risks, reduced cardiorespiratory fitness, cardiovascular stability problems, and lifestyle factors were accumulated. LASSO-based weight analysis also supported this interpretation. In the final analysis, dyslipidemia, low cardiorespiratory fitness, hypertension, ECG abnormalities, and habitual alcohol consumption were the main items related to the CSSI-based high-risk classification.

Low cardiorespiratory fitness applying the age- and sex-specific reference values of the KOSHA H-43–2021 and the Canadian Public Health Association accounted for a high proportion in additive CSSI, and was repeatedly selected in the bootstrap stability analysis. This suggests that VO₂max-based cardiorespiratory fitness indicators can partially reflect the functional reserve required for confined space work. In confined spaces, wearing protective equipment, heat burden, limited ventilation, the possibility of oxygen shortage, emergency response, etc. may occur at the same time, so a greater metabolic and cardiopulmonary functional burden may occur than in a general working environment. Therefore, the VO₂max-related information obtained from the screening test may be used as an auxiliary indicator for estimating the functional reserve before working in a confined space. Low cardiorespiratory fitness does not mean a simple decrease in exercise ability, but can be interpreted as an indicator that physiological reserve may be limited in the context of continuing work while wearing protective equipment, exposure to heat burden, emergency escape, or rescue response [40,41].

Methodologically, this study is differentiated in that it calculated the LASSO weight based on the combination of risk factors observed in the actual data, not the method by which the researcher gave a subjective score in advance [42]. This approach does not treat all risk factors with the same weight, but allows relatively larger weights to be assigned to items more closely related to the CSSI-based high-risk classification. In the final additive CSSI, dyslipidemia, low cardiorespiratory fitness, abnormal resting heart rate, hypertension, and ECG abnormalities showed high weights. This shows that not only metabolic risk but also cardiopulmonary function, cardiovascular stability, and lifestyle factors should be considered together in the selection of confined space work suitability. However, the weight of CSSI should be understood as the relative weight of risk factors involved in CSSI-based high-risk classification, not as a numerical value of specialist judgment. At rest, the heart rate may fluctuate depending on temporary factors such as tension, fati‌‌gue, and caffeine intake at the time of the test, while high blood pressure reflects a relatively persistent cardiovascular burden, so it may have a separate meaning when judging the risk of acute events under load conditions [43].

CSSI is not a tool for the same purpose as the Work Ability Index (WAI). WAI is useful for evaluating workers’ overall work ability and subjective health status, but it is difficult to sufficiently capture specific physiological criteria required for high-risk tasks due to the large proportion of self-report items. On the other hand, CSSI includes objective test data such as lung function, blood tests, electrocardiogram, and cardiorespiratory fitness, and reflects items that are directly linked to confined space-specific risks such as the possibility of oxygen deficiency, burden of wearing protective equipment, and metabolic reserve. Therefore, CSSI can help to supplement the self-report-centered assessment and to consider the relative weight of objective test results and risk factors together in the pre-placement screening assessment.

The practical significance of this study is that it presented a preliminary framework that can classify workers who need attention before working in confined spaces in stages using standard health examination and functional test data. Academically, it is meaningful in that health information from different areas is integrated into a single indicator and the contribution of each risk factor is differentiated based on data. In practice, it can help determine which workers should be connected to additional evaluation first in a workplace where specialist evaluation resources are limited. Large-scale workplaces have room to operate a precision evaluation system centered on specialists by referring to strict standards such as NFPA 1582, but it is difficult to conduct the same level of evaluation at all times in small and medium-sized workplaces. The CSSI may serve as a structured screening tool to help prioritize workers who require further specialist evaluation using standard health examination data. In workplaces with limited access to specialized medical resources, CSSI can help prioritize health care and specialist referrals before confined-space work assignment. However, this is not to replace specialist evaluation but should be interpreted as a screening aid to more efficiently utilize limited resources.

The CSSI may also help guide health management based on individual risk-factor profiles beyond a simple fit/unfit classification. This enables interventions based on workers’ specific risk profiles [44,45]. For example, workers with high blood pressure and low VO₂max are not simply excluded from work but can be subject to specific cardiovascular management and aerobic exercise programs. This approach suggests the possibility of extending fitness-for-duty assessment toward an occupational health strategy focused on risk-factor improvement and preventive management, rather than simply determining fit or unfit status.

## Limitations

Several limitations should be considered in this study. First, the sample size was relatively small (n = 111), the CSSI-defined unsuitable group was small (n = 6), and the number of workers classified as unsuitable in the independent specialist assessment was also limited (n = 7). This low frequency of unsuitability may be related to the healthy worker effect, because workers undergoing pre-placement evaluation in actual industrial settings may have relatively better health status. Although this limitation reduced the statistical power for extensive subgroup analyses, leave-one-out cross-validation and bootstrap stability analysis showed that major risk factors were repeatedly selected in the limited sample. However, these findings should be interpreted as preliminary evidence.

Second, the study utilized a cross-sectional design, which prevents causal inference regarding the progression of health risks. Third, while the cutoffs for risk factors were based on established guidelines, VO₂max may vary depending on the test protocol and calculation method [46]. In this study, low cardiorespiratory fitness was defined by applying age- and sex-specific reference values from KOSHA H-43–2021 and the Canadian Public Health Association; however, further validation using directly measured VO₂max or a standardized exercise testing protocol is needed in future studies. Fourth, functional test results may be influenced by transient factors such as daily condition or fatigue.

Finally, since this model is composed mainly of individual physiological suitability and health risk factors, it did not directly reflect the environmental conditions at the time of actual work. For example, real-time field monitoring data such as oxygen concentration, temperature, humidity, and concentrations of harmful gases were not included in this analysis. In future studies, it is necessary to expand this approach to a dynamic safety management model that can reflect risk changes during work beyond pre-work screening evaluation by using personal health risk information and field environmental sensor data together [47].

## Conclusion

In this study, the Confined-Space Suitability Index (CSSI) was developed to systematically identify workers who need additional specialist fitness-for-duty assessments before confined space work. CSSI was calculated by integrating metabolic risk, cardiorespiratory fitness, cardiovascular stability, and lifestyle factors that can be obtained from general health examination and functional test data using a LASSO-based weighted summation method. In the final analysis, dyslipidemia, low cardiorespiratory fitness, abnormal resting heart rate, hypertension, and ECG abnormalities were identified as major weighted items constituting the CSSI-based high-risk classification. CSSI is not a tool that replaces the final fitness-for-duty judgment of a specialist, and can be used as a screening index to first identify workers who need additional evaluation in an environment where occupational health resources are limited and to assist in health management and referral decisions before work assignment. In the future, it is necessary to perform external validation of CSSI using various industrial sites and larger worker datasets.

## Supporting information

S1 File Supplementary tables. This file contains Supplementary Table S1 and Supplementary Table S2. Supplementary Table S1 presents the risk-factor profiles of workers classified as unsuitable by the revised CSSI rule-based category. Supplementary Table S2 presents the bootstrap stability analysis of LASSO-selected risk factors.
(DOCX)
