# Supplementary material for: Development of a Confined-Space Suitability Index (CSSI) using Least Absolute Shrinkage and Selection Operator (LASSO) regression: a pilot study for structured fitness-for-duty screening
Source: PLoS One. 2026 Jun 2;21(6):e0350740. doi: 10.1371/journal.pone.0350740 (PMC13229319; doi:10.1371/journal.pone.0350740)
Supplement: S1 File — This file contains Supplementary Table S1 and Supplementary Table S2. Supplementary Table S1 presents the risk-factor profiles of workers classified as unsuitable by the revised CSSI rule-based category. Supplementary Table S2 presents the bootstrap stability analysis of LASSO-selected risk factors. (DOCX) [file pone.0350740.s001.docx]

**Supplementary Table S1. Risk-factor profiles of workers classified as unsuitable by the revised CSSI rule-based category**

| Participant index | Contributing risk factors | Total number of risk factors | Additive CSSI score |
| --- | --- | --- | --- |
| 21 | Hypertension, Dyslipidemia, Elevated liver enzymes, Hearing loss, Abnormal ECG, Low cardiorespiratory fitness | 6 | 91.1 |
| 27 | Obesity, Hypertension, Dyslipidemia, Elevated liver enzymes, Low cardiorespiratory fitness, Habitual alcohol consumption | 6 | 86.0 |
| 45 | Obesity, Hypertension, Dyslipidemia, Elevated liver enzymes, Abnormal ECG, Low cardiorespiratory fitness, Habitual alcohol consumption | 7 | 100.0 |
| 53 | Obesity, Hypertension, Dyslipidemia, Abnormal pulmonary function, Low cardiorespiratory fitness, Habitual alcohol consumption | 6 | 86.9 |
| 95 | Diabetes, Dyslipidemia, Abnormal resting heart rate, Hearing loss, Low cardiorespiratory fitness, Significant smoking history | 6 | 74.0 |
| 110 | Dyslipidemia, Elevated liver enzymes, Abnormal pulmonary function, Abnormal ECG, Low cardiorespiratory fitness, Habitual alcohol consumption | 6 | 83.7 |

Participants listed were classified as unsuitable by the revised CSSI rule-based category. Additive CSSI scores were calculated using normalized positive LASSO-derived weights and scaled to a 0–100 range. CSSI, Confined-Space Suitability Index; ECG, electrocardiogram.

**Supplementary Table S2. Bootstrap stability analysis of LASSO-selected risk factors**

| Risk factor | Selection frequency | Coefficient median | Coefficient Q1 | Coefficient Q3 | Mean coefficient |
| --- | --- | --- | --- | --- | --- |
| Dyslipidemia | 0.981 | 1.134 | 0.784 | 1.436 | 1.093 |
| Low cardiorespiratory fitness | 0.976 | 0.867 | 0.601 | 1.102 | 0.839 |
| Hypertension | 0.952 | 0.8 | 0.51 | 1.077 | 0.793 |
| Habitual alcohol consumption | 0.928 | 0.606 | 0.344 | 0.871 | 0.625 |
| Abnormal electrocardiographic finding | 0.85 | 0.456 | 0.132 | 0.73 | 0.468 |
| Elevated liver enzymes | 0.732 | 0.316 | 0 | 0.726 | 0.422 |
| Abnormal pulmonary function | 0.685 | 0.165 | 0 | 0.494 | 0.268 |
| Hearing loss | 0.666 | 0.176 | 0 | 0.471 | 0.26 |
| Abnormal resting heart rate | 0.618 | 0.613 | 0 | 0.83 | 0.482 |
| Diabetes | 0.418 | 0 | 0 | 0 | 0.025 |
| Significant smoking history | 0.369 | 0 | -0.102 | 0 | -0.079 |
| Obesity | 0.302 | 0 | 0 | 0 | 0.048 |
| Age | 0.126 | 0 | 0 | 0 | -0.003 |
| Anemia | 0.108 | 0 | 0 | 0 | -0.015 |
| Male sex | 0.001 | 0 | 0 | 0 | 0 |

Selection frequency indicates the proportion of bootstrap resamples in which each variable had a non-zero coefficient. Bootstrap analysis was performed using 1,000 resampling iterations; 998 valid iterations were retained after excluding resamples containing only one outcome class.
